# Hyperglycemia Leads to BMSC Impaired Osteogenesis, Enhanced Adipogenesis, and Altered Metabolism

**DOI:** 10.1002/jcb.70090

**Published:** 2026-04-25

**Authors:** Suzanna Shirazi, Ezaldeen Esawi, Zeyad D. Nassar, Stan Gronthos, Dimitrios Cakouros

**Affiliations:** ^1^ Mesenchymal Stem Cell Laboratory, School of Pharmacy and Biomedical Sciences, College of Health Adelaide University South Australia Australia; ^2^ Solid Tumour Program, Precision Medicine Theme South Australian Health and Medical Research Institute Adelaide South Australia Australia; ^3^ South Australian ImmunoGENomics Cancer Institute Adelaide University Adelaide South Australia Australia

**Keywords:** adipogenesis, BMSC, bone, hyperglycemia, mesenchymal/stromal stem cells, metabolites, osteogenesis

## Abstract

Diabetes is a major risk factor for osteoporosis, which negatively impacts bone health, but the mechanisms underlying the effects of hyperglycemia on bone marrow mesenchymal/stromal cells (BMSC) are not fully understood. This study investigated how high glucose levels influence BMSC differentiation, proliferation, viability, and metabolism. The results demonstrated that high glucose inhibits osteogenesis in human BMSC, as evidenced by reduced alkaline phosphatase activity, impaired calcium deposition, and downregulation of key osteogenic genes (RUNX2, ALP). Conversely, high glucose conditions promoted adipogenesis, characterized by increased percentage of cells with lipid droplets, and upregulation of adipogenic genes (PPARγ2, CEBPα, AdipoQ), suggesting a shift towards fat cell differentiation. Furthermore, BMSC cultured in high glucose showed decreased proliferation, elevated DNA damage, increased oxidative stress, enhanced apoptosis and senescence, particularly in later passages, highlighting the negative impact of hyperglycemia on BMSC viability. Metabolomic profiling of osteogenic and adipogenic differentiation in normal and high glucose conditions revealed key metabolic shifts, with nicotinamide adenine dinucleotide (NAD+) and l‐glutamate/α‐ketoglutarate (α‐KG) identified as critical metabolites driving these processes. Supplementation with NAD+ and α‐KG in high glucose conditions significantly enhanced ALP activity. These findings suggest that high glucose promotes adipogenesis at the expense of osteogenesis, exacerbating cellular damage and accelerating aging in BMSC. The identification of NAD+ and α‐KG as key regulators in this process provides new insights into the metabolic mechanisms behind impaired bone health in diabetes and highlights potential therapeutic avenues to counteract these detrimental effects to better manage diabetes‐related bone diseases.

## 1 Introduction

Diabetes is a chronic disease characterized by high blood glucose levels, greatly impacting various organ systems, including the heart, blood vessels, eyes, kidneys, and bone homeostasis. In recent years, the complicated interplay between metabolic disorders and skeletal health has gained increasing attention within the field of biomedical research. Among these disorders, hyperglycemia, a hallmark of diabetes mellitus, stands out not only for its systemic impact on overall body physiology but also for its profound implications on skeletal health and function [1]. Bone is an insulin‐responsive organ that plays a role in whole‐body energy metabolism. Elevated glucose levels have been associated with disrupted bone homeostasis, leading to compromised bone quality, increased fracture risk and osteoporosis, which is a common age‐related disease of bone fragility [2, 3]. Bone healing in patients with poorly controlled diabetes mellitus is notably impaired and associated with conditions like diabetic osteopathy [4]. Several factors have been identified as potential contributors, including growth hormone dysfunction, deficiencies in growth factors, poor vascularization, neuropathy, advanced glycation end products (AGEs) and systemic and local inflammation [5, 6]. At the cellular microenvironment level, increased adipogenicity in bone marrow [7], impaired osteoblastic activity [8], deregulated endochondral ossification [9], increased cellular senescence and apoptosis [10] are key drivers of the bone pathologies associated with diabetes. Understanding how dietary glucose affects the growth, function, and metabolic dynamics of bone marrow cells is crucial for revealing the mechanisms underlying skeletal complications associated with metabolic disease, as up to now, these mechanisms by which high glucose levels affect bone metabolism remain elusive.

Bone marrow mesenchymal/stromal stem cells (BMSC) are the central players in bone regeneration and maintenance [11]. Bone marrow serves as a reservoir for BMSC, which are essential for tissue repair, hematopoietic support and bone remodeling [12]. BMSCs are multipotent progenitors residing within the bone marrow microenvironment, exhibit remarkable plasticity and respond dynamically to metabolic cues. They are capable of differentiating into various cell types, including adipocytes, chondrocytes, and osteoblasts. These cells play a crucial role in maintaining bone homeostasis and are central to bone regeneration [13]. MSCs differentiate into osteoblasts and adipocytes through distinct transcription factors. RUNX2 is key in osteogenesis, activating osteoblast‐specific genes like osteocalcin (OCN), osteopontin (OPN), and alkaline phosphatase (ALP), while suppressing adipogenesis. In contrast, adipogenesis is regulated by CEBPα and PPARγ, which control leptin and adiponectin, promoting lipid storage and metabolic regulation. PPARγ is crucial for lipid accumulation and insulin sensitivity in mature fat cells, with CEBPα collaborating to drive adipocyte formation [14]. Evidence suggests that BMSC undergo significant metabolic adaptations in response to hyperglycemic conditions, altering their differentiation potential and functional properties [15].

Metabolomics, the comprehensive study of small molecules within a biological system, offers a detailed view of cellular metabolic changes. Glycolysis is thought to support the “stemness” of BMSC [16], but recent research shows that aerobic glycolysis primarily drives their differentiation, whereas inhibiting glycolysis can reduce the osteogenic potential of bone marrow mesenchymal progenitors [17]. The key metabolites produced from glycolysis, the tricarboxylic acid (TCA) cycle, and related processes have a profound impact on gene expression, chromatin structure, and cellular differentiation. Nicotinamide adenine dinucleotide (NAD+) acts as a cofactor for histone deacetylases and sirtuins, impacting gene expression. High NAD+ levels have been shown to enhance stem cell differentiation and maintain stemness, whereas NAD+ depletion impairs differentiation and accelerates cellular aging [18]. α‐Ketoglutarate (α‐KG) is a cofactor for Jumonji C‐domain containing histone demethylases, which remove methyl groups from histones, influencing chromatin structure and gene expression. High α‐KG concentrations promote differentiation in stem cells, while lower levels help maintain stemness [19]. The present study explores the complex relationship between high glucose levels and BMSC differentiation. Using human BMSC, we examined the cellular mechanisms through which high glucose levels influence proliferation, functional capacities, and metabolic adaptations.

## 2 Methods

### 2.1 Cell Culture

BMSC were isolated from the posterior iliac crests of 3 adult volunteers, 2 females and 1 male in their 20's, with informed consent and ethical approval protocol number 940911A from the Royal Adelaide Hospital. Bone marrow cells were pooled and diluted to 50 mL with Hanks buffer (or blocking buffer for flow cytometry). To isolate mononuclear cells, 7.5 mL of marrow was layered onto 3 mL of Lymph prep and centrifuged at 400*g* for 30 min at room temperature without brake. The mononuclear cell layer was collected, washed twice in Hank's buffer containing 5% FCS and resuspended in FCS. Final cell counts and yields were recorded, and cells were cryopreserved at 2 × 10^7 cells per ampoule. STRO‐1^+^ BMSC were isolated by magnetic activated cell sorting [12], and were maintained in human complete media containing Dulbecco's Modified Eagle Medium (DMEM) with low (normal) glucose level with 1000 mg/L glucose (Cat#11885084‐500 mL, Thermofisher) or high glucose level with 4500 mg/L glucose (Cat#11995065‐500 mL, Thermofisher), supplemented with 10% fetal bovine/calf serum (FCS) (Cat#AU‐FBS/PG, CellSera, Rutherford, NSW, AUS), 1 mM sodium pyruvate (Cat#S8636‐100mL, Sigma Aldrich), 10 mM HEPES buffer (Cat#H0887‐100mL, Sigma Aldrich), 2mM l‐glutamine (Cat#G7513‐100mL, Sigma Aldrich), 100 μM ascorbate‐2‐phosphate (Cat# A8960‐5G, Sigma Aldrich) and 100 U/mL penicillin, 100 μ/mL streptomycin (P/S) (Cat#P4333‐100 mL, Sigma Aldrich) as previously described [20].

### 2.2 Oxidative Stress Assay

Human BMSC were cultured in normal or high glucose media, plated in 96‐well plates (2 × 10³ cells/well), and treated with 100 µL of 25 µM CM‐H2DCFDA (Cat#D399, Thermo Fisher Scientific) to assess oxidative stress. After incubation, cells were exposed to 100 µL of 600 µM ABAP (except controls) (Cat# 440914, Sigma Aldrich), and kinetic fluorescence was measured at 538 nm using a Synergy/HTX reader.

### 2.3 DNA Damage Assay

Human BMSC were cultured in normal or high glucose media, plated in 8‐well chamber slides (5 × 10³ cells/well), and incubated for 2–3 days. Cells were fixed, permeabilized (Cat# 9036‐19‐5, Sigma Aldrich), and treated overnight with primary antibody (Anti‐phospho‐Histone H2A.X) (Cat# 05‐636, Merck). After staining with goat anti‐mouse Alexa 488 (ThermoFisher Cat# A‐11008) and DAPI (Cat# H‐2000, Vector Laboratories, Newark, California, United States), slides were mounted with anti‐fade and DNA damage visualized using an Olympus IX81 inverted fluorescence microscope, and images were captured using CellSens light microscopy software. ImageJ software was used to quantify DNA damage levels (green fluorescence), normalized to the non‐irradiated control. Three fields of view were imaged per well, with a minimum of 50 cells per field.

### 2.4 Apoptosis Assay

BMSC were cultured in normal and high glucose media (5 × 10⁴ cells in 6‐well plates). Seventy‐two hours before analysis, 5 µM Dox/Etoposide was added to apoptosis controls, and 70% ethanol induced necrosis in positive controls. After collection, cells were stained with Annexin V (Alexa Fluor 488 conjugate, Thermofisher) and 7‐AAD (Bekham Coulter), then analyzed by flow cytometry (FACSCanto II) and BD FACSCanto II clinical software. Analysis was performed using three human donors per cohort.

#### 2.4.1 Senescence Assay

BMSC (5 × 10^4^) were cultured in six‐well plate for 48 h in normal and high glucose level media. The β‐galactosidase (β‐Gal) staining kit was used to determine senescence percentage according to the manufacturer's specifications (cell signaling technology). Senescence cells (blue) were quantified relative to total cell number by visualization using an Olympus IX53 light microscopy at ×200 magnification and CellSens software. SA‐*β*‐gal‐positive cells were counted using ImageJ software and normalized to the total cell count. Analysis was performed using three donors in each cohort in triplicate.

### 2.5 Cell Proliferation Assay

Human BMSC were cultured in normal and high glucose media in 96‐well plates (2 × 10³ cells/well). After 5 days, cells were treated with BrdU for 24 h. Proliferation was assessed using the BrdU cell proliferation ELISA (Roche), following the manufacturer's instructions. The absorbance was measured at 450 nm using a Biotek Synergy HTX Multimode plate reader. Analysis was performed using three donors from each cohort in triplicate.

### 2.6 In Vitro Differentiation Assays

We previously reported the conditions for the induction of human BMSC to develop a mineralized bone matrix or adipocyte formation in vitro [21]. Human BMSC were cultured in normal or high glucose media under control, osteogenic (0.1 µM Dexamethasone, 2.6 mM Potassium Phosphate), or adipogenic (60 µM Indomethacine, 0.1 µM Dexamethasone) conditions. After 1 week of osteogenic induction, mineralization was assessed via ALP activity (405 nm) and ALP staining. After 3 weeks, extracellular calcium was measured and normalized to DNA content and also visualized by alizarin red staining as previously described [22]. After 4 weeks of adipogenic induction, lipid formation was quantified by Nile red fluorescence, normalized to DAPI, and visualized as previously described [21, 22].

Real‐time polymerase chain reaction analysis: Total RNA was isolated from 5 × 10⁴ BMSC using TRIzol reagent (Invitrogen), and cDNA synthesized with Superscript IV reverse transcriptase (Invitrogen). Gene expression was analyzed by real‐time PCR using RT2 SYBR Green/Rox master mix (SABiosciences) on a QuantStudio 3 Real‐Time PCR System. PCR reactions were performed in triplicate, validated by melt curve analysis, and gene expression changes were calculated relative to β‐actin using the 2^−ΔCt^ method. Primer details are in Table S1.

Metabolomics sample preparation and analysis: human BMSC were cultured for 7 and 21 days in normal or high glucose media under control, osteogenic, or adipogenic conditions. A total of 48 samples were processed by the Australian Wine Research Institute, South Australia, where 110 compounds were detected using HILIC LC‐MS. Compound identification was based on internal and external spectral databases. Data from this study is available at https://doi.org/10.21228/M8FZ5G and https://dev.metabolomicsworkbench.org:22222/data/DRCCMetadata.php?Mode=Study&StudyID=ST003704&Access=PdlD5696 website.

### 2.7 PCA and Heatmap Generation

Counts were log‐transformed and Pareto‐scaled. PCA was applied to visualize clustering with condition‐based ellipses. Heatmaps of metabolite expression were created with hierarchical clustering and row scaling. All analyses were performed in R using tidyverse and pheatmap packages (Version 1.0.12) (https://cran.r-project.org/web/packages/pheatmap/index.html).

### 2.8 NAD+ and α‐Ketoglutarate Level Confirmation Tests

Human BMSCs were cultured in normal and high glucose media in T25 flasks. For NAD+ quantification, 1 × 10⁵ cells were pelleted, washed, and homogenized in NAD+ or NADH extraction buffer. Cell lysates and standards were transferred to a 96‐well plate, and fluorescence was measured at 530 nm (excitation) and 585 nm (emission). The NAD+/NADH ratio was calculated using the NAD^+^/NADH Assay kit's formula (Sigma‐Aldrich, Catalog No. MAK460). For α‐KG quantification, 1 × 10⁶ cells were pelleted, washed, and homogenized. Lysates and standards were added to a 96‐well plate, and α‐KG levels were quantified by measuring fluorescence at 530–570 nm (excitation) and 590–600 nm (emission). Concentration was calculated using the alpha‐ketoglutarate assay kit formula (Cell Biolabs, Catalog No. MET5131).

### 2.9 Metabolic Pathways Analysis

Metabolites were categorized based on their ratio (> 1 or < 1) between adipogenic/osteogenic and control groups, as well as normal versus high glucose. KEGG IDs were determined using MetaboAnalyst, and pathways were analyzed for significance (Raw *p* value < 0.05, FDR < 1). Pathways meeting these criteria were further examined. NAD+ and α‐KG, selected for their relevance, were chosen for detailed investigation.

#### 2.9.1 Statistical Analysis

Statistical *p* value differences between the high glucose‐mediated BMSC and normal glucose‐mediated BMSC were determined using the two‐way ANOVA with Sidak's multiple comparison test (*p < 0.05, **p < 0.01, ***p < 0.001, ****p < 0.0001). All statistical analyses were conducted using GraphPad Prism v8.0.0.

## 3 Results

### 3.1 High Glucose Levels Inhibit Osteogenesis and Osteogenic Gene Expression

**Figure 1:**
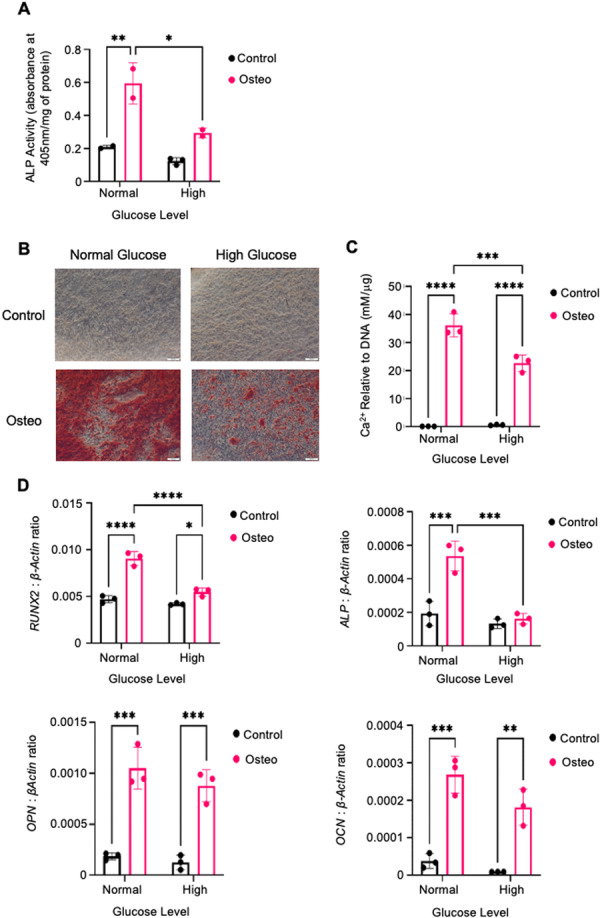
High glucose levels inhibit osteogenesis and osteogenic gene expression. BMSC from three donors were cultured in normal or high glucose media with control or osteogenic conditions. (A) ALP activity was quantified and normalized to total protein. (B) Alizarin red staining visualized matrix formation at 4× magnification. (C) Extracellular calcium was quantified using the Calcium Arsenazo III reagent, normalized to DNA content. (D) qPCR was used to measure RUNX2, OCN, ALP, and OPN gene expression relative to β‐actin. Mean values ± SEM, *n* = 3 independent donors (*p < 0.05, **p < 0.01, ***p < 0.001, ****p < 0.0001).

To investigate the effect of high glucose on osteogenesis, BMSC from three donors were cultured in normal or high glucose media with control or osteogenic supplements. ALP activity, a marker of early osteogenic differentiation, was significantly reduced under high glucose conditions (Figure 1A). Similarly, levels of Alizarin red‐stained mineral deposits and extracellular calcium levels decreased under high glucose osteogenic conditions (Figure 1B,C). Additional evidence data indicated that expression of key osteogenic genes (RUNX2, ALPL) was significantly downregulated under high glucose conditions, in both control and osteogenic media (Figure 1D). These data indicate that high glucose levels impair BMSC‐mediated mineralization.

### 3.2 High Glucose Levels Promote Adipogenesis and Adipogenic Gene Expression

**Figure 2:**
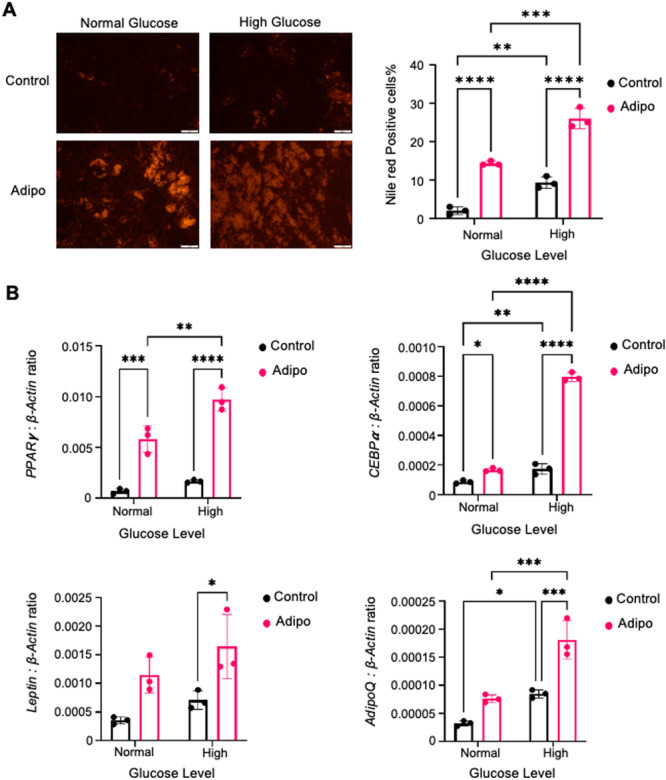
High glucose levels promote adipogenesis and adipogenic gene expression. BMSC from three donors were cultured in normal or high glucose media with control or adipogenic conditions. (A) Nile Red and DAPI‐stained lipid droplets and fat cells observed under fluorescence microscopy at 10× magnification (B) PCR was used to measure PPARγ2, CEBPα, Leptin, and AdipoQ relative to β‐actin. Mean ± SEM, *n* = 3 donors (*p < 0.05, **p < 0.01, ***p < 0.001, ****p < 0.0001).

To assess high glucose effects on adipogenic differentiation, BMSC were cultured in normal or high glucose media with control or adipogenic supplements. Nile Red staining showed increased lipid accumulation in BMSC under high glucose conditions, with larger fat droplets and more fat cells compared to normal glucose (Figure 2A). Gene expression analysis of adipogenic markers, PPARγ2, CEBPα, and AdipoQ, revealed significant upregulation in high glucose conditions, indicating enhanced adipogenesis (Figure 2B). These findings suggest that high glucose promotes adipogenesis in BMSC, favoring the adipogenic pathway over osteogenesis.

### 3.3 High Glucose Levels Promote Cell Death and Reduce Cellular Proliferation in Human BMSC

**Figure 3:**
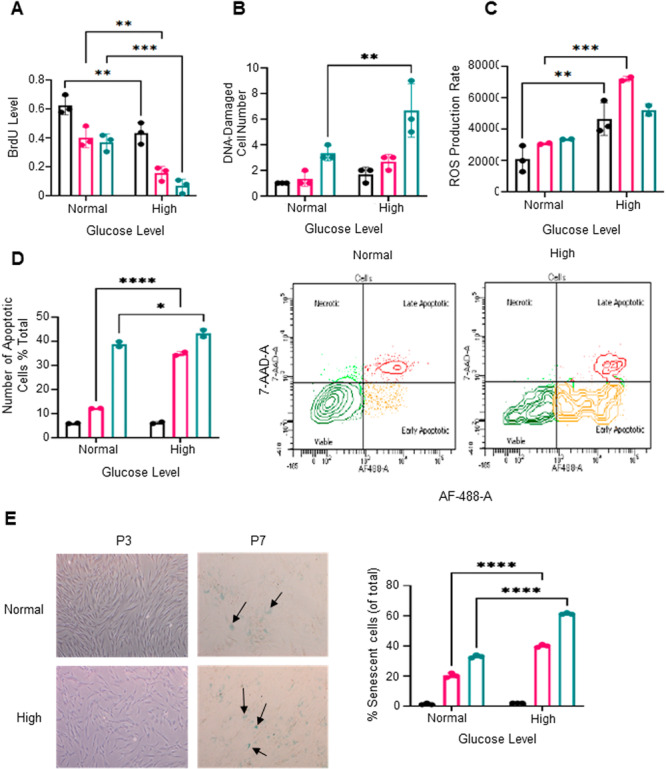
High glucose levels drive cell death and reduce cellular proliferation in human BMSC. BMSC were passaged and tested at early (cell passage 3; black), intermediate (cell passage 5; red) and late (cell passage 7; green) passages for: (A) BrdU incorporation (proliferation), (B) DNA damage, (C) ROS production (oxidative stress), (D) apoptotic cells (early/late), and (E) β‐galactosidase activity (blue) in cells are pointed with arrows at 4× magnification, with the percentage of β‐galactosidase‐positive cells calculated in senescence cells. Mean values n = 3 donors, (*p < 0.05, **p < 0.01, ***p < 0.001, ****p < 0.0001).

The impact of high glucose on BMSC viability was investigated by culturing cells under normal and high glucose conditions across successive cell passages, early (P3), intermediate (P5) and late (P7). Cellular growth, DNA damage, oxidative stress, apoptosis, and senescence were assessed. Cell proliferation was found to decrease with passage number and was significantly lower in high glucose conditions, as measured by BrdU incorporation, indicating inhibited proliferation compared to normal glucose conditions (Figure 3A). DNA damage was observed to be significantly higher in high glucose conditions, assessed by γ‐H2AX foci, with greater damage observed in later passages (P7), indicating increased genomic instability over time (Figure 3B). Given the reduction in cell growth and increase in DNA damage we postulated that this could be partly driven by the high glucose levels increasing reactive oxygen species, which can damage DNA. High glucose levels also increased reactive oxygen species (ROS) in BMSC across all cell passages, implying that elevated oxidative stress contributes to cellular damage and dysfunction (Figure 3C). Apoptosis levels were also higher in BMSC under high glucose conditions, especially in later passages (P5 and P7), as measured by annexin V staining. This correlated with reduced proliferation and increased DNA damage, highlighting the harmful effects of high glucose on cell viability (Figure 3D). Similarly, cellular senescence was higher in BMSC cultured in high glucose at all passages, indicating that high glucose accelerates cellular aging (Figure 3E), as measured by β‐galactosidase activity. Collectively, these data indicate that high glucose impairs BMSC function by promoting adipogenesis over osteogenesis, reducing proliferation, increasing DNA damage, oxidative stress, apoptosis, and accelerating senescence, highlighting its adverse effects on BMSC viability and function.

### 3.4 Metabolic Profiling of Adipogenic and Osteogenic Groups Under Normal and High Glucose Conditions: Insights From PCA and Heat Map Analysis

**Figure 4:**
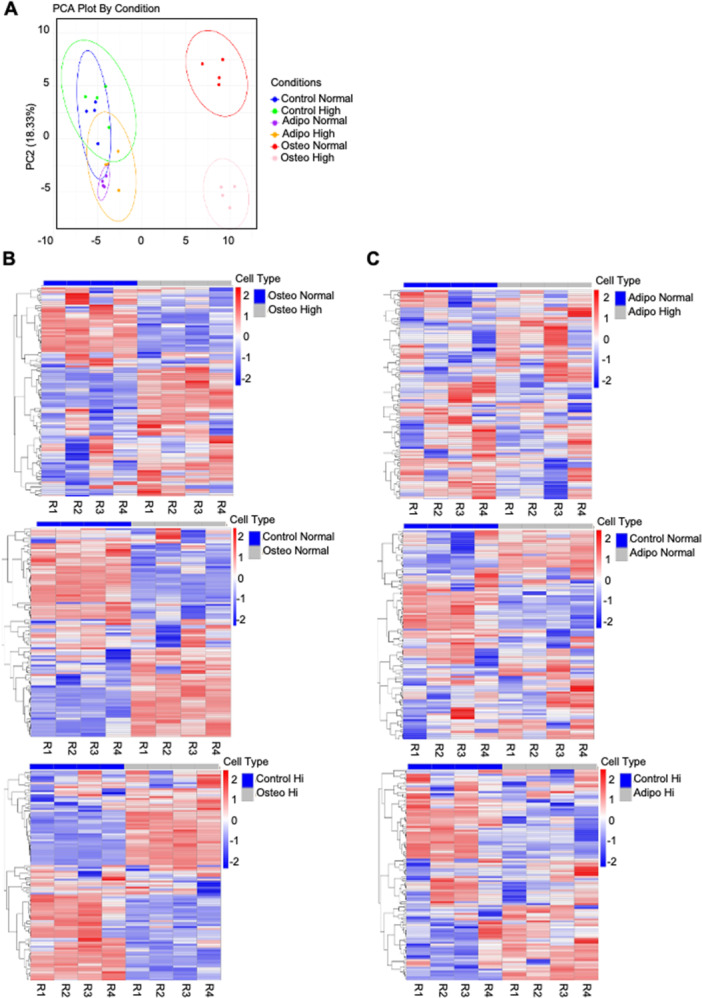
PCA and heat map analysis reveal glucose‐dependent differentiation profiles of BMSC under osteogenic and adipogenic induction. BMSCs from three donors were cultured under normal and high glucose media with control, osteogenic, or adipogenic conditions. Metabolomics analysis revealed: (A) PCA Scores Plot showing separation between osteogenic groups and clustering of adipogenic, osteogenic, and control samples. (B) Osteogenic) and (C) adipogenic heat maps displaying metabolite abundance, with red indicating upregulated and blue downregulated metabolites, comparing osteogenic versus control and adipogenic versus control groups across normal and high glucose conditions.

To explore the metabolic differences between adipogenic and osteogenic groups under normal and high glucose conditions, we identified the most significant metabolites present in each condition and employed Principal Component Analysis (PCA) and heat map analysis. PCA revealed clear metabolic distinctions, with separate clustering of osteogenic groups under normal and high glucose conditions. Metabolites from adipogenic and osteogenic groups differed significantly from controls, with the loadings plot identifying key metabolites driving the clustering (Figure 4A). The heat map showed distinct metabolic profiles, with red indicating upregulated metabolites and blue indicating downregulated ones. The data revealed strong differentiation between osteogenic and control groups, as well as adipogenic and control groups, with minimal difference between normal and high glucose conditions. Hierarchical clustering identified consistent metabolic changes, particularly between osteogenic/adipogenic and control groups (Figure 4B–C), suggesting glucose levels had a lesser impact on the overall metabolic profile.

### 3.5 Identification of Key Metabolic Pathways and Metabolites in Adipogenic and Osteogenic Conditions Under Normal and High Glucose

Metabolites were categorized based on their ratio (> 1 or < 1) relative to control groups to identify key metabolites associated with adipogenic, osteogenic, and glucose conditions. KEGG IDs for these metabolites were retrieved via MetaboAnalyst and used in Pathway Analysis. Pathway analysis identified significant pathways (*p *< 0.05, FDR < 1) that were altered between adipogenic and osteogenic groups, as well as between normal and high glucose conditions (Tables S2 and S3). Upon reviewing the properties of the selected metabolites, NAD+ and l‐glutamate were selected for further investigation due to their significant changes in the analyzed pathways (Tables S4 and S5). The level of NAD+ increases in adipogenic condition while l‐glutamate decreases in osteogenic conditions compared to control. l‐glutamate, converted to α‐KG, plays a key role in cell differentiation, while NAD+ and α‐KG impact cellular metabolism and gene transcription, making them crucial for this study. Overall, this analysis highlighted key metabolic shifts associated with different conditions and set the stage for further investigation into the roles of NAD+ and α‐KG in regulating adipogenic and osteogenic processes.

### 3.6 Effect of α‐KG and NAD+ on BMSC Properties in High Glucose Conditions

**Figure 5:**
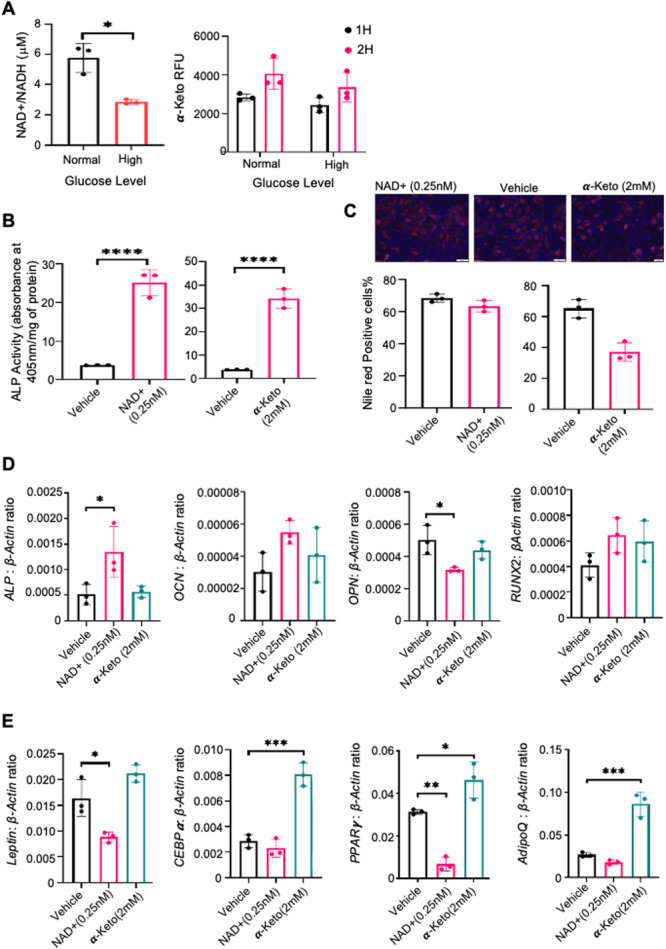
*α*‐Ketoglutarate and NAD+ induction change osteogenic and adipogenic properties of human BMSC in high glucose levels. BMSC from three donors were cultured under normal and high glucose conditions. (A) NAD+ and α‐KG levels were measured in high glucose media. α‐KG levels were measured kinetically (1H, 2H). (B) Cells were induced with NAD+ (0.25 nM) or α‐KG (2 mM) under high glucose, and ALP activity and (C) fat content (Nile Red and DAPI staining) were assessed at 10× magnification. (D) qPCR of cDNA measured osteogenic gene expression (OCN, ALPL, RUNX2, OPN) and (E) adipogenic gene expression (Leptin, PPARγ2, CEBPα, AdipoQ) relative to β‐actin. Mean values ± SEM, *n* = 3 independent MSC donors, (*p < 0.05, **p < 0.01, ***p < 0.001, ****p < 0.0001).

High glucose levels were found to significantly decrease NAD+ and α‐KG levels (Figure 5A). Supplementing with NAD+ (0.25 nM) or α‐KG (2 mM) for 4 days increased ALP activity, indicating enhanced osteogenic potential (Figure 5B). Nile Red and DAPI staining showed reduced lipid accumulation with both NAD+ and α‐KG supplementation (Figure 5C). Gene expression analysis revealed that NAD+ upregulated osteogenic markers (OCN, ALP, RUNX2) but not OPN and downregulated adipogenic genes (Leptin, PPARγ2, CEBPα, AdipoQ). Furthermore, α‐KG promoted osteogenesis, but with less significant gene expression changes (Figure 5D,E). Interestingly, even though α‐KG reduced adipogenesis, it seemed to increase adipogenic gene expression. Overall, the results clearly show that NAD+ promotes osteogenesis and osteogenic gene expression. Similarly, α‐KG also promotes osteogenesis, however, no significant transcription changes were seen at the time point examined.

## 4 Discussion

Our findings show that high glucose alters BMSC differentiation, proliferation, and viability, impairing osteogenesis, promoting adipogenesis, and increasing cellular stress. These results emphasize the impact of hyperglycemia on bone health and metabolic disorders like osteoporosis and obesity.

Our data indicate that high glucose levels inhibit osteogenic differentiation in BMSC, as evidenced by significantly reduced ALP activity and impaired extracellular calcium deposition. ALP activity was notably suppressed in BMSC cultured under high glucose conditions, suggesting a negative impact on osteoblastic differentiation. Similarly, the decreased calcium deposition in high glucose‐exposed BMSC points to impaired mineralization, a hallmark of osteogenesis. These results are consistent with previous studies demonstrating that hyperglycemia disrupts osteogenesis and bone mineralization, potentially contributing to the increased fracture risk observed in diabetic patients [23, 24, 25].

Moreover, our gene expression analysis revealed that key osteogenic transcription factors and markers, including RUNX2 and ALPL, were downregulated in high glucose conditions. Suppression of these markers in high glucose conditions may explain the observed inhibition of osteogenesis.

In contrast to its effects on osteogenesis, high glucose levels favored adipogenesis in BMSC. Nile red staining demonstrated significantly increased lipid accumulation in BMSC cultured under high glucose conditions, suggesting enhanced adipogenic differentiation. This increase in adipocyte‐like cells under high glucose conditions was accompanied by upregulation of adipogenic markers such as PPARγ2, CEBPα and AdipoQ, further supporting the notion that high glucose promotes adipogenesis. The preference for adipogenic over osteogenic differentiation under high glucose conditions may contribute to the pathological accumulation of fat tissue observed in metabolic disorders like type 2 diabetes mellitus (TIIDM), where skeletal health is often compromised.

High glucose exposure shifts differentiation towards adipogenesis, likely due to a metabolic environment favoring lipogenesis. This supports findings that high glucose or hyperinsulinemia promotes adipocyte differentiation over osteoblast differentiation [26, 27]. The imbalance between osteogenesis and adipogenesis may contribute to insulin resistance and metabolic syndrome, complicating diabetes management. Reduced osteoblast differentiation lowers osteocalcin production, impairing insulin sensitivity and disrupting energy homeostasis, promoting metabolic disorders like insulin resistance and TIIDM [28].

High glucose impaired BMSC proliferation and viability, especially at later passages. This reduction in proliferative capacity was linked to increased DNA damage, oxidative stress, apoptosis, and cellular senescence. These results suggest that high glucose not only affects differentiation but also accelerates cellular aging, reducing long‐term viability. The combined effects of reduced proliferation, DNA damage, and senescence highlight the negative impact of high glucose on BMSC function, potentially hindering tissue repair and regeneration in diabetic conditions [29, 30].

Moreover, the metabolic analysis provided additional insights into the underlying mechanisms driving the observed cellular changes. The metabolism of glucose, fatty acids, and amino acids supplies the energy necessary for the differentiation of BMSC. Metabolic pathways can also be modified by other lifestyle factors such as environment and biological conditions, which in turn can influence BMSC differentiation. This emphasizes the importance of metabolism in determining BMSC fate and suggests that targeting metabolic processes may provide potential therapeutic approaches for conditions related to BMSC dysfunction, such as obesity and osteoporosis [31].

In this current study, PCA and heat map analysis revealed significant metabolic distinctions between osteogenic and adipogenic conditions, with clear separation between the two groups regardless of glucose levels. While metabolic shifts were evident between osteogenic and adipogenic groups, glucose levels appeared to have a less pronounced effect on overall metabolic profiles. One likely reason is that the time points of 1 and 3 weeks of cell culture used in the metabolomic screen likely missed the maximum fluctuations in metabolites driven by glucose. This is supported by the significant effect high glucose had on NAD+ when we examined it at earlier time points, showing a significant decrease NAD+ is essential in mitochondrial function and plays crucial roles in energy metabolism as a coenzyme in multiple redox reactions in major catabolic pathways, promoting organismal longevity and stimulating the osteogenesis of BMSC [32, 33]. Furthermore, α‐KG is a key intermediate in the TCA cycle and is recognized as a reprogramming metabolite that serves as a cofactor for various chromatin‐modifying enzymes. Studies have shown that supplementing osteoporotic mice with α‐KG can reverse the osteoporotic phenotype by increasing bone growth osteogenesis [34, 35].

Hyperglycemia has been shown to significantly impact the metabolism of BMSC, particularly through its effects on α‐KG [36]. Under high glucose conditions, the unusual increased availability of glucose can lead to elevated production of ROS, which subsequently alters cellular metabolism and diminishes α‐KG levels [36]. Reduced α‐KG levels can impair the differentiation potential of BMSC and enhance adipogenesis, thereby contributing to altered bone metabolism and increased risk of osteoporosis [37].

Results from our study revealed key metabolic pathways significantly altered between adipogenic and osteogenic conditions under both normal and high glucose conditions. Particularly, NAD+ and α‐KG were identified as central metabolites in these pathways. NAD+ and α‐KG are crucial in cellular metabolism, and their altered levels under high glucose conditions may contribute to the observed shifts in differentiation. Our study's significant NAD+ decrease aligns with Yen et al. [38], who found that high glucose intake rapidly depletes NAD+, shifting MSC lineage commitment from osteogenesis to adipogenesis by reducing SIRT1 activity. This highlights the harmful effects of high glucose on MSC function and bone health [38].

To explore the potential of reversing the negative effects of high glucose on BMSC function, we examined the impact of supplementing BMSC with NAD+ and α‐KG. Our results indicate that both metabolites had positive effects on osteogenic differentiation under high glucose conditions. NAD+ supplementation enhanced ALP activity, suggesting that NAD+ may restore some aspects of osteogenic potential in hyperglycemic conditions.

Similarly, α‐KG supplementation increased ALP activity. Wang et al. [35] found that α‐KG enhanced bone mass and regeneration in aged mice by reducing H3K9me3 and H3K27me3 accumulation in BMSC, improving proliferation and osteogenic potential [35]. Moreover, we have previously shown that the α‐KG‐dependent enzymes, Tet family of proteins, specifically Tet2 promotes osteogenesis and a decrease in its activity is associated with osteoporosis [20]. While NAD+ downregulated adipogenic markers (Leptin and PPARγ), α‐KG upregulated them (CEBPα, PPARγ and adipoQ), suggesting a complex role in regulating differentiation. Further investigation is needed, as only one time point was examined. In summary, our study shows that high glucose levels negatively impact BMSC differentiation, proliferation, and viability by inhibiting osteogenesis and promoting adipogenesis. High glucose also exacerbates cellular damage, oxidative stress, and senescence. Metabolic changes in NAD+ and α‐KG influence BMSC differentiation.

## Author Contributions

Suzanna Shirazi performed experiments, collected, analyzed, and interpreted all of the study data and was a contributor in writing the manuscript. Ezaldeen Esawi and Zeyad D. Nassar provided reagents, analyzed and interpreted the data and were contributors in writing the manuscript. Dimitrios Cakouros performed experiments, analyzed and interpreted the data, and was a contributor in writing the manuscript. Stan Gronthos analyzed and interpreted the data, provided funding for the study, and was a contributor in writing the manuscript. All authors contributed to the creation of the study's concept and design, and read and approved the final manuscript.

## Ethics Statement

The research was prospectively reviewed and approved by the Royal Adelaide Hospital Human Ethics Committee under protocol number 940911A.

## Consent

Written informed consent was obtained from each patient for the use of their samples for research purposes only and the publication of this manuscript. Their personal data has not been divulged.

## Conflicts of Interest

The authors declare no conflicts of interest.

## Supporting information

Supporting Table 1:

Supporting Table 2:

Supporting Table 3:

Supporting Table 4:

Supporting Table 5:

## Data Availability

The data that support the findings of this study are openly available in “Metabolomics Workbench” at https://dev.metabolomicsworkbench.org:22222/data/DRCCMetadata.php?Mode=Study&StudyID=ST003704&Access=PdlD5696, reference number (ST003704).
