# Supplementary material for: Hyperglycemia Leads to BMSC Impaired Osteogenesis, Enhanced Adipogenesis, and Altered Metabolism
Source: J Cell Biochem. 2026 Apr 25;127(4):e70090. doi: 10.1002/jcb.70090 (PMC13109826; doi:10.1002/jcb.70090)
Supplement: Supplementary file 1 — Supporting Table 1: [file JCB-127-e70090-s004.docx]

**Supplementary Table 1**. Sequences of primers used in quantitative real-time reverse transcription PCR (qRT-PCR).

| **Gene** |  | **Sequence** |
| --- | --- | --- |
| β‐actin | Forward primer | 5′‐gatcattgctcctcctgagc‐3′ |
|  | Reverse primer | 5′‐gtcatagtccgcctagaagcat‐3′ |
| CEBPα | Forward primer | 5′-tgtatacccctggtgggaga-3′ |
|  | Reverse primer | 5′-tcataactccggtccctctg-3′ |
| PPARγ2 | Forward primer | 5′-ttctggatttcactatggagttcatgc-3′ |
|  | Reverse primer | 5′-attatgagacatccccactgcaagg-3′ |
| Leptin | Forward primer | 5′-gaaccctgtgcggattcttgt-3′ |
|  | Reverse primer | 5′-tccatcttggataaggtcaggat-3′ |
| OPN | Forward primer | 5′‐acatccagtaccctgatgctacag‐3′ |
|  | Reverse primer | 5′‐gtgggtttcagcactctggt‐3′ |
| OCN | Forward primer | 5′‐atgagagccctcacactcctcg‐3′ |
|  | Reverse primer | 5′‐gtcagccaactcgtcacagtcc‐3′ |
| ALPL | Forward primer | 5′‐ccagtctcatctcctgaccc-3′ |
|  | Reverse primer | 5′‐ggtggtcttggagtgagtga-3′ |
| Runx2 | Forward primer | 5′‐gtggacgaggcaagagtttca‐3′ |
|  | Reverse primer | 5′‐catcaagcttctgtctgtgcc‐3′ |
