# Supplementary material for: Hyperglycemia Leads to BMSC Impaired Osteogenesis, Enhanced Adipogenesis, and Altered Metabolism
Source: J Cell Biochem. 2026 Apr 25;127(4):e70090. doi: 10.1002/jcb.70090 (PMC13109826; doi:10.1002/jcb.70090)
Supplement: Supplementary file 2 — Supporting Table 2: [file JCB-127-e70090-s001.docx]

**Supplementary Table 2.** Metabolic pathways that were significantly increased in BMSC cultured under adipogenic condition compared to control group (Raw p < 0.05, FDR <1).

| Pathways | Total | Expected | Hits | Raw p | Holm adjust | FDR | Impact |
| --- | --- | --- | --- | --- | --- | --- | --- |
| Nicotinate and nicotinamide metabolism | 15 | 0.089641 | 2 | 0.003203 | 0.26908 | 0.2164 | 0.23465 |
| Pantothenate and CoA biosynthesis | 19 | 0.11355 | 2 | 0.005152 | 0.42764 | 0.2164 | 0.00714 |
| Alanine, aspartate and glutamate metabolism | 28 | 0.16733 | 2 | 0.011075 | 0.90811 | 0.31009 | 0.3101 |
| Arginine and proline metabolism | 38 | 0.22709 | 2 | 0.019964 | 1 | 0.41925 | 0.02385 |
| D-Glutamine and D-glutamate metabolism | 6 | 0.035857 | 1 | 0.035383 | 1 | 0.59443 | 0 |
