# Supplementary material for: Hyperglycemia Leads to BMSC Impaired Osteogenesis, Enhanced Adipogenesis, and Altered Metabolism
Source: J Cell Biochem. 2026 Apr 25;127(4):e70090. doi: 10.1002/jcb.70090 (PMC13109826; doi:10.1002/jcb.70090)
Supplement: Supplementary file 3 — Supporting Table 3: [file JCB-127-e70090-s002.docx]

**Supplementary Table 3.** Metabolic pathways that were significantly decreased in BMSC cultured under osteogenic conditions compared to control group (Raw p < 0.05, FDR <1).

| Pathways | Total | Expected | Hits | Raw p | Holm adjust | FDR | Impact |
| --- | --- | --- | --- | --- | --- | --- | --- |
| Arginine biosynthesis | 14 | 0.065073 | 2 | 0.003203 | 0.13792 | 0.090533 | 0.11675 |
| Histidine metabolism | 16 | 0.074369 | 2 | 0.002156 | 0.17891 | 0.090533 | 0 |
| Glutathione metabolism | 28 | 0.13015 | 2 | 0.006611 | 0.54209 | 0.13883 | 0.02675 |
| Alanine, aspartate and glutamate metabolism | 28 | 0.13015 | 2 | 0.006611 | 0.54209 | 0.13883 | 0.42068 |
| Arginine and proline metabolism | 38 | 0.17663 | 2 | 0.012023 | 0.96186 | 0.20199 | 0.086 |
| Aminoacyl-tRNA biosynthesis | 48 | 0.22311 | 2 | 0.018865 | 1 | 0.26411 | 0 |
| Nitrogen metabolism | 6 | 0.027888 | 1 | 0.027612 | 1 | 0.28992 | 0 |
| D-Glutamine and D-glutamate metabolism | 6 | 0.027888 | 1 | 0.027612 | 1 | 0.28992 | 0.5 |
| Valine, leucine and isoleucine biosynthesis | 8 | \| 0.037185 \| \| --- \| | 1 | 0.036669 | 1 | 0.30802 | 0 |
| Taurine and hypotaurine metabolism | 8 | 0.037185 | 1 | 0.036669 | 1 | 0.30802 | 0.42857 |
