# Supplementary material for: Hyperglycemia Leads to BMSC Impaired Osteogenesis, Enhanced Adipogenesis, and Altered Metabolism
Source: J Cell Biochem. 2026 Apr 25;127(4):e70090. doi: 10.1002/jcb.70090 (PMC13109826; doi:10.1002/jcb.70090)
Supplement: Supplementary file 4 — Supporting Table 4: [file JCB-127-e70090-s003.docx]

|  |
| --- |
| **Supplementary Table 4.** Metabolites that repeated the most between Adipogenic/ Control BMSC samples.   \| Adipo/Cont Increased Pathways \| Metabolites \| \| --- \| --- \| \| Nicotinate and Nicotinamide Metabolism \| **L-Aspartate, NAD+** \| \| Pantothenate and CoA Biosynthesis \| **L-Aspartate, Pantothenate** \| \| Alanine, Aspartate and Glutamate Metabolism \| **L-Aspartate, 4-Aminobutanoate** \| \| Arginine and Proline Metabolism \| **4-Aminobutanoate, D-Proline** \| \| D-Glutamine and D-Glutamate Metabolism \| **D-Glutamine** \| |

|  |
| --- |
|  |
|  |
|  |
|  |
|  |
|  |
|  |
|  |
