# Supplementary material for: Hyperglycemia Leads to BMSC Impaired Osteogenesis, Enhanced Adipogenesis, and Altered Metabolism
Source: J Cell Biochem. 2026 Apr 25;127(4):e70090. doi: 10.1002/jcb.70090 (PMC13109826; doi:10.1002/jcb.70090)
Supplement: Supplementary file 5 — Supporting Table 5: [file JCB-127-e70090-s005.docx]

**Supplementary Table 5.** Metabolites that repeated the most between Osteogenic/ Control BMSC samples.

| \| \| Osteo/Cont Decreased Pathways \| Metabolites \| \| --- \| --- \| \| Arginine Biosynthesis \| **L-Aspartate, L-Glutamate** \| \| Histidine Metabolism \| **L-Aspartate, L-Glutamate** \| \| Glutathione Metabolism \| **L-Glutamate, 5-Oxoproline** \| \| Alanine, Aspartate and Glutamate Metabolism \| **L-Aspartate, L-Glutamate** \| \| Arginine and Proline Metabolism \| **L-Glutamate, Phosphocreatine** \| \| Aminoacyl-Trna Biosynthesis \| **L-Aspartate, L-Glutamate** \| \| Nitrogen Metabolism \| **L-Glutamate** \| \| D-Glutamine and D-Glutamate Metabolism \| **L-Glutamate** \| \| Valine, Leucine and Isoleucine Biosynthesis \| **2-Oxobutanoate** \| \| Taurine And Hypotaurine Metabolism \| **Taurine** \| \| \| --- \| --- \| --- \| --- \| --- \| --- \| --- \| --- \| --- \| --- \| --- \| --- \| --- \| --- \| --- \| --- \| --- \| --- \| --- \| --- \| --- \| --- \| --- \| |
| --- | --- | --- | --- | --- | --- | --- | --- | --- | --- | --- | --- | --- | --- | --- | --- | --- | --- | --- | --- | --- | --- | --- | --- |
